# Emergency Department and Observation Revisits and the Hospital Readmission Reduction Program

**DOI:** 10.1001/jamanetworkopen.2025.24135

**Published:** 2025-07-30

**Authors:** Bimatshu Pyakuryal, Prasun Pudasainee, Alisha Sharma, Narendra Veerapaneni, Sanjay Singh, Sneha Nagavally, Barbara Slawski, Pinky Jha, Sanjay Bhandari

**Affiliations:** 1 Division of General Internal Medicine, Medical College of Wisconsin, Milwaukee; 2 Department of Medicine, Medical College of Wisconsin, Milwaukee

## Abstract

This retrospective cohort study analyzes the burden of emergency department and observation revisits on overall 30-day hospital revisits across Hospital Readmission Reduction Program–targeted conditions.

## Introduction

The burden of emergency department (ED) and observation revisits following an inpatient admission has gained increasing attention, particularly for its impact on reimbursement policies under the Hospital Readmission Reduction Program (HRRP).^1,2,3^ Currently, the HRRP’s 30-day readmission metric considers only inpatient readmissions, excluding patients who return to the ED or require observation stays.^4^ This study aims to provide a comprehensive, multiyear analysis of the relative burden of ED and observation revisits on overall 30-day hospital revisits across HRRP-targeted conditions.

## Methods

We conducted a retrospective cohort study of Medicare beneficiaries aged 65 years and older who were hospitalized at Froedtert Hospital and Medical College of Wisconsin Network (both in Milwaukee, Wisconsin) between 2015 and 2023 with primary diagnoses of HRRP-targeted conditions. These conditions include acute myocardial infarction, chronic obstructive pulmonary disease, heart failure, pneumonia, coronary artery bypass graft, and total hip or knee arthroplasty. We collected patient demographics and clinical characteristics, including age, sex, race and ethnicity, and comorbidities, along with hospital utilization data such as total index inpatient admissions, and 30-day hospital revisits (inpatient readmissions, ED revisits, and observation revisits). Data on race and ethnicity are included in this study because they are known key determinants of health outcomes including readmissions.

Descriptive statistics were used to summarize patient demographics and hospital utilization patterns. The dataset was extracted from the Epic Clarity reporting database and was analyzed using R statistical software version 4.1.3 (R Project for Statistical Computing) and SQL Server Management Studio version SSMS 2017 (Microsoft). The study was approved by the institutional review board at the Medical College of Wisconsin ensuring compliance with ethical standards; informed consent was not needed because the data were deidentified, in accordance with 45 CFR §46. It follows the STROBE reporting guideline.

## Results

Table 1 shows the number of total index inpatient admissions, along with 30-day inpatient readmissions, ED revisits, and observation revisits for each HRRP-targeted condition. Of the total 76 936 index admissions, 11 633 (15.1%) resulted in 30-day total hospital revisits. Of these revisits, 8456 (72.7%) were inpatient readmissions, 2301 (19.8%) were ED revisits, and 876 (7.5%) were observation revisits. Pneumonia accounted for the highest burden of 30-day hospital revisits (5144 of 11 633 visits [44.2%]), followed by heart failure (3360 visits [28.9%]) and chronic obstructive pulmonary disease (2196 visits [18.9%]).

**Table 1.** Descriptive Characteristics of Index Inpatient Admissions and 30-Day Hospital Revisits

30-d Hospital revisits	Index inpatient admissions/total No. of 30-d hospital revisits (%)						
	AMI (n = 5875)	CABG (n = 810)	COPD (n = 15 483)	HF (n = 17 765)	PNA (n = 35 278)	THA or TKA (n = 1725)	Total (N = 76 936)^a^
30-d Inpatient readmissions	489/695 (70.4)	90/119 (75.6)	1603 (73.0)	2429 (72.3)	3760 (73.1)	85 (71.4)	8456 (72.7)^b^
30-d Emergency department revisits	139/695 (20.0)	21/119 (17.6)	430 (19.6)	649 (19.3)	1039 (20.2)	23 (19.3)	2301 (19.8)^c^
30 d Observation unit revisits	67/695 (9.6)	8/119 (6.7)	163 (7.4)	282 (8.4)	345 (6.7)	11 (9.2)	876 (7.5)^d^

Abbreviations: AMI, acute myocardial infarction; CABG, coronary artery bypass graft; COPD, chronic obstructive pulmonary disease; HF, heart failure; HRRP, hospital readmissions reduction program; PNA, pneumonia; THA, total hip arthroplasty; TKA, total knee arthroplasty.

^a^ Refers to total index inpatient admissions of all HRRP-targeted conditions (AMI, CABG, COPD, HF, PNA, and THA or TKA).

^b^ Total 30-day inpatient readmissions of all HRRP targeted conditions.

^c^ Refers to total 30-day ED revisits of all HRRP-targeted conditions.

^d^ Total 30-day observation revisits of all HRRP-targeted conditions.

Table 2 summarizes the characteristics of unique patients with 30-day hospital revisits. The mean (SD) age recorded at their first index inpatient admission was 77.56 (8.42) years. Of these, 3361 (52.1%) were female, 64 (1.0%) were Hispanic, 1148 (17.8%) were Non-Hispanic Black, 5086 (78.8%) were Non-Hispanic White, and 154 (2.4%) were other races and ethnicities. A total of 678 (10.5%) had dual Medicare-Medicaid coverage, and the mean (SD) Elixhauser Comorbidity Index score was 5.41 (2.71).

**Table 2.** Descriptive Characteristics of Unique Patients With an Index Inpatient Admission Diagnosis of AMI, CABG, COPD, HF, PNA, THA, or TKA Who Had 30-Day Hospital Revisits

Characteristic	Patients, No. (%)						
	AMI (n = 442)	CABG (n = 75)	COPD (n = 1174)	HF (n = 1855)	PNA (n = 2839)	THA or TKA (n = 68)	Total (N = 6453)
Age at index admission, mean (SD) [range], y	77.51 (8.67) [65-99]	77.53 (7.58) [65-99]	76.24 (7.66) [65-98]	79.09 (8.78) [65-103]	77.09 (8.34) [65-102]	76.15 (7.74) [65-101]	77.56 (8.42) [65-103]
Sex							
Female	194 (43.9)	22 (29.3)	649 (55.3)	994 (53.6)	1464 (51.6)	38 (55.9)	3361 (52.1)
Male	248 (56.1)	53 (70.7)	525 (44.7)	861 (46.4)	1375 (48.4)	30 (44.1)	3092 (47.9)
Race and ethnicity^a^							
Hispanic	6 (1.4)	1 (1.3)	8 (0.7)	21 (1.1)	28 (1.0)	0	64 (1.0)
Non-Hispanic Black	43 (9.7)	7 (9.3)	226 (19.3)	368 (19.8)	498 (17.5)	6 (8.8)	1148 (17.8)
Non-Hispanic White	379 (85.7)	63 (84.0)	920 (78.4)	1427 (76.9)	2236 (78.8)	61 (89.7)	5086 (78.8)
Other^b^	14 (3.2)	4 (5.3)	20 (1.7)	39 (2.1)	76 (2.7)	1 (1.5)	154 (2.4)
Missing	0	0	0	0	1 (<0.1)	0	1 (<0.1)
Medicare or Medicaid coverage	30 (6.8)	9 (12.0)	151 (12.9)	202 (10.9)	282 (9.9)	4 (5.9)	678 (10.5)
Elixhauser Comorbidity Index score, mean (SD) [range]^c^	4.90 (2.71) [0.00-15.00]	6.53 (2.63) [1.00-16.00]	5.54 (2.67) [0.00-16.00]	6.26 (2.54) [0.00-16.00]	4.87 (2.68) [0.00-15.00]	4.85 (2.45) [0.00-11.00]	5.41 (2.71) [0.00-16.00]

Abbreviations: AMI, acute myocardial infarction; CABG, coronary artery bypass graft; COPD, chronic obstructive pulmonary disease; HF, heart failure; PNA, pneumonia; THA, total hip arthroplasty; TKA, total knee arthroplasty.

^a^ Race and ethnicity were self-reported.

^b^ Other includes American Indian or Alaska Native, Asian, Native Hawaiian or Other Pacific Islander, multiracial, unknown, and any other race not otherwise specified.

^c^ Calculated at the time of data extraction.

## Discussion

Using 9 years of data from a large academic center, this retrospective cohort study found that more than one-quarter of all 30-day hospital revisits occur through the ED and observation unit. Given the high burden of ED and observation revisits, our findings support scholars’ call for HRRP metric reform by incorporating total hospital revisits, rather than solely focusing on inpatient readmissions.^1,2,3^

Study limitations included a single-center design and nonstratification by individual years. In addition, the same patients may have had multiple revisits within the span of 30 days. Furthermore, since the study included data only up to 2023, any hospital revisits that occurred afterward were not captured.

Multicenter studies are necessary to improve generalizability and validate our findings. Further research is warranted to investigate the short-term and long-term outcomes of hospital revisits through ED and observation stays compared with those with inpatient readmissions.

## Notes

Supplement.
Data Sharing Statement
