# Error in Byline

**DOI:** 10.1001/jamanetworkopen.2025.39062

**Published:** 2025-09-26

In the Research Letter titled “Emergency Department and Observation Revisits and the Hospital Readmission Reduction Program,”^1^ published July 30, 2025, there was an error in the byline. Coauthor Sneha Nagavally, PhD, was omitted. This article has been corrected.^1^
