# Supplementary material for: Emergency Department and Observation Revisits and the Hospital Readmission Reduction Program
Source: JAMA Netw Open. 2025 Jul 30;8(7):e2524135. doi: 10.1001/jamanetworkopen.2025.24135 (PMC12311697; doi:10.1001/jamanetworkopen.2025.24135)
Supplement: Supplement. — Data Sharing Statement [file jamanetwopen-e2524135-s001.pdf]

Data Sharing Statement

Pyakuryal. Emergency Department and Observation Revisits and the Hospital Readmission Reduction Program. *JAMA Netw Open*. Published July 30, 2025.
doi:10.1001/jamanetworkopen.2025.24135

Data
Data available: No
